# Cellular plasticity and metastasis in breast cancer: a pre- and post-malignant problem

**DOI:** 10.20517/2394-4722.2019.26

**Published:** 2019-06-13

**Authors:** Jacob M. Smigiel, Sarah E. Taylor, Benjamin L. Bryson, Ilaria Tamagno, Kelsey Polak, Mark W. Jackson

**Affiliations:** 1 Department of Pathology, Case Western Reserve University School of Medicine, Cleveland, OH 44106, USA.; 2 Case Comprehensive Cancer Center, Case Western Reserve University, Cleveland, OH 44106, USA.

**Keywords:** Cell plasticity, pre-malignant plasticity, breast cancer, epithelial-mesenchymal transition/cancer stem cell, metastasis

## Abstract

As a field we have made tremendous strides in treating breast cancer, with a decline in the past 30 years of overall breast cancer mortality. However, this progress is met with little affect once the disease spreads beyond the primary site. With a 5-year survival rate of 22%, 10-year of 13%, for those patients with metastatic breast cancer (mBC), our ability to effectively treat wide spread disease is minimal. A major contributing factor to this ineffectiveness is the complex make-up, or heterogeneity, of the primary site. Within a primary tumor, secreted factors, malignant and pre-malignant epithelial cells, immune cells, stromal fibroblasts and many others all reside alongside each other creating a dynamic environment contributing to metastasis. Furthermore, heterogeneity contributes to our lack of understanding regarding the cells’ remarkable ability to undergo epithelial/non-cancer stem cell (CSC) to mesenchymal/CSC (E-M/CSC) plasticity. The enhanced invasion & motility, tumor-initiating potential, and acquired therapeutic resistance which accompanies E-M/CSC plasticity implicates a significant role in metastasis. While most work trying to understand E-M/CSC plasticity has been done on malignant cells, recent evidence is emerging concerning the ability for pre-malignant cells to undergo E-M/CSC plasticity and contribute to the metastatic process. Here we will discuss the importance of E-M/CSC plasticity within malignant and pre-malignant populations of the tumor. Moreover, we will discuss how one may potentially target these populations, ultimately disrupting the metastatic cascade and increasing patient survival for those with mBC.

## INTRODUCTION: THE MORTALITY OF METASTATIC BREAST CANCER

Breast cancer (BC) is the most commonly diagnosed cancer among women, and the second leading cause of cancer related deaths in women^[1]^. The statistics highlight the importance of metastasis in BC mortality: in patients with distant metastasis, 5-year survival rates are only 22% (13% at 10 years), compared to 90% for patients with local disease^[2]^. Furthermore, for those patients with metastatic BC (mBC), there are currently no effective treatment options. Understanding how BC cells escape the primary tumor, spread to distant organs, initiate outgrowth at a distant site, and then developing therapies to target those metastatic processes remains a significant clinical challenge. Our understanding of the metastatic cascade has increased in recent years: cells must degrade the extracellular matrix (ECM) surrounding them, extravasate into the circulatory or lymphatic system and circulate throughout the body, intravasate into the new organ tissue, and regain their proliferative capacity^[3–5]^. Yet, the molecular mechanisms driving each of these processes, all of which are important for a successful metastatic event, remain unresolved.

A complicating factor to our understanding of BC metastasis is the heterogeneous milieu of the primary tumor site, or tumor micro-environment (TME), which is comprised of epithelial, endothelial, immune, and stromal cells. It is important to note that the epithelial populations can be subdivided into two distinct groups, malignant and pre-malignant. The malignant population has completed the transformation process through loss of tumor suppressive mechanisms and a gain of oncogenic signaling, via genetic mutation or sustained growth factor or cytokine signaling. Conversely, pre-malignant refers to a spectrum of points on the path towards transformation. Pre-malignant cells drift further from normalcy as they acquire mutations and engage aberrant signaling. If intact, a tumor suppressive response may be engaged to halt the transformation process, however if lost, the cell may progress to a fully transformed endpoint. The complex cellular composition within the TME results in a network of secreted factors and ECM proteins, which also profoundly influence metastatic potential^[6–9]^. Numerous TME factors can drive epithelial cells to undergo epithelial-mesenchymal transition (EMT) and acquire cancer stem cell (CSC) properties, which we refer to as epithelial-mesenchymal (E-M)/CSC plasticity^[10–14]^. Seminal work has defined E-M/CSC plasticity as an important step in metastasis and is often investigated from the perspective of a malignant population. However, malignant cells are not the only populations capable of undergoing E-M/CSC reprogramming. Recent evidence has demonstrated a remarkable ability of pre-malignant epithelial cells to take on a more invasive phenotype able to intravasate and disseminate to secondary sites following signaling cues from the TME^[15,16]^. Here, we discuss the challenges of targeting various cell populations and the signaling pathways that contribute to the cellular plasticity driving mBC. Importantly, we will explore the impact of pre-malignant cells escaping senescence by undergoing E-M/CSC reprogramming to gain invasive, metastatic, and tumor-initiating properties. Identifying determinants of metastasis, such as E-M/CSC plasticity, and advancing our ability to target the drivers of plasticity will have a significant impact on survival for those with mBC.

## SETTING THE STAGE: HETEROGENEITY WITHIN THE PRIMARY SITE

A major challenge in dealing with BC is the heterogeneity that accompanies it. With three distinct clinical subtypes, estrogen and progesterone receptor positive (ER+/PR+), human epidermal growth factor receptor 2 positive (HER2+), and triple negative breast cancer (TNBC; negative for ER, PR, HER2 expression), finding the proper treatment options can be difficult^[17–19]^. Therapies targeting ER/PR hormone signaling via selective estrogen receptor modulators (SERMs) or aromatase inhibitors (i.e., Tamoxafin and Arimidex) have significantly improved patient survival and have made this subtype more manageable. Likewise, HER2+ tumors can be treated with antibodies or kinase inhibitors targeting HER2 signaling (i.e., Trastuzamab and Lapatinib) and are often met with success provided the disease is caught early; the efficacy of these therapies drastically decreases with late stage, metastatic HER2+ BC^[20–23]^. Conversely, TNBC currently lacks a targeted therapy tailored to a specific driver oncogene and is most often treated with cytotoxic chemotherapies. Patients with TNBC exhibit an increased risk of metastatic dissemination resulting in higher clinical stage at diagnosis and lower disease-free survival compared to patients with non-TNBC cancers^[24]^. Much like metastatic HER2+ BC, metastatic TNBC is not effectively treated, highlighting the need for better therapies targeting those cells which progress beyond the primary site and are ultimately responsible for patient mortality and morbidity. Further challenges in treating BC involve the heterogeneous nature of the tumor cells themselves, a phenomenon often referred to as intra-tumoral heterogeneity (ITH)^[25]^. Evidence suggests that across a panel of human cancers, including breast, increased ITH correlates with decreased overall survival, and therapy resistance^[26,27]^. Furthermore, high ITH inversely correlates with low tumor infiltrating lymphocytes, which are often associated with increased patient survival^[28–36]^.

The path to ITH is complex and involves a series of genetic and epigenetic events throughout the transformation process which permit normal human mammary epithelial cells (HMEC) to develop into fully malignant cancer cells^[37–44]^. Progress in RNA and DNA sequencing technologies have helped shape the evolutionary picture of HMEC; losing tumor suppressor function (TP53 mutations or RB loss) and acquiring oncogenic drivers (MYC, HER2, or CCND1 amplification or PIK3CA mutations)^[45–49]^. Genetic alterations lead to the expansion of a pre-malignant population which progressively acquires additional genetic and epigenetic changes until one or more cells become fully transformed^[50,51]^. These additional mutations are numerous, and genomic profiling has found a wide variety of changes in copy number, chromatin alterations, chromosomal rearrangements, and point mutations throughout the genome from single cell sequencing of bulk tumor tissue in TNBC^[36,52,53]^. Not only does this dynamic process of transformation alter the cancer cell itself, but transforming cells have a substantial impact on the surrounding environment. Evidence suggests that the accumulation of mutations within epithelial cells can lead to a dysregulated secretory network, including a number of inflammatory cytokines linked to poor prognosis, therapy failure, and disease recurrence (IL-6, IL-8, TGF-β, CCL2, TNF-α, IL-17 and others)^[54–59]^. This dysregulated secretory network in turn, changes the cellular composition of the TME, leading to a reciprocal cross-talk between non-cancerous stromal cells and the transforming epithelial cells. Overall, this demonstrates the immense complexity of the tumor, as the heterogeneity described above culminates in a highly diverse TME, with an array of cell types, secreted factors, and structural make up.

Importantly, not all epithelial cells that begin the transformation process reach full malignancy. As a cell senses aberrant activation of signaling pathways/gene induction, it may enact intact tumor suppressive mechanisms, resulting in senescence^[60–66]^. Senescence is a major growth-inhibiting and tumor-suppressive barrier that must be bypassed *in vivo* during transformation en-route to tumor development^[63,67–73]^. Large senescent cell populations can be found at various stages of tumor development, further contributing to tumor heterogeneity. Remarkably, an investigation by Cotarelo *et al*.^[74]^ was able to identify the presence of senescent cells in approximately 83.7% of the human invasive breast carcinomas examined, suggesting their involvement throughout the transformation process and as tumors evolve and progress. Since the 129 tumors surveyed in this study were from untreated patients, the origin of senescence within these invasive BC is an *in vivo* physiological response.

**Figure 1.**
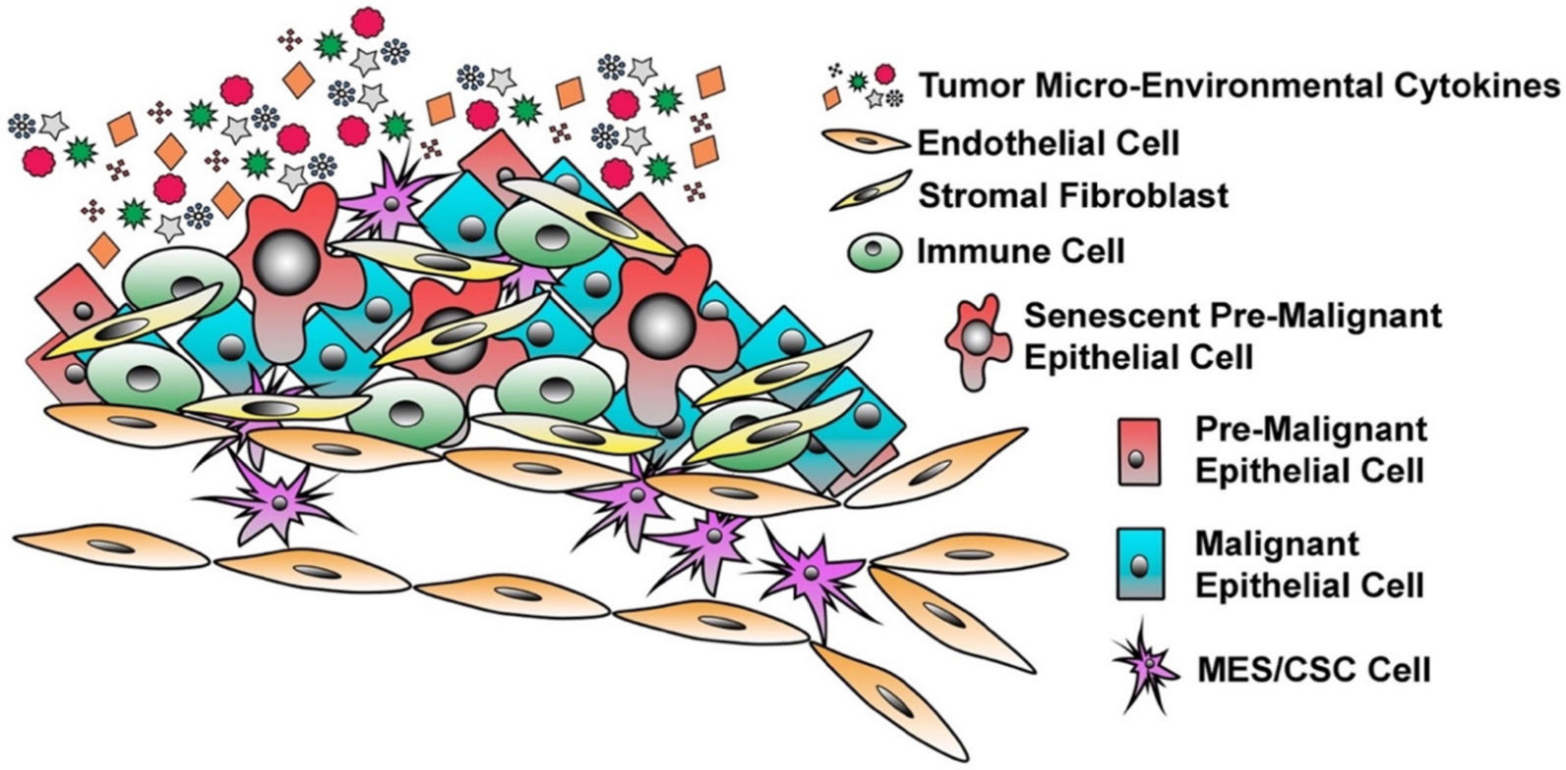
The Heterogeneity of Breast Cancer. Breast cancer is a heterogeneous disease with a highly dynamic tumor micro-environment (TME). Within the primary site, one can observe the presence of epithelial cells that have undergone E-M/CSC plasticity, malignant epithelial cells, pre-malignant cells, senescent epithelial cells, stromal fibroblasts, infiltrating immune cells, and endothelial cells. The presence of all of these diverse cell types results in a distinct and complex milieu of secreted factors within the TME that influence, tumor progression, disease recurrence, and cell plasticity

Long thought inert, bystanders within the tumor, senescent cells have gained considerable interest for their potential impact on the tumor as a whole. Despite being growth-arrested, senescent cells remain viable, metabolically active, and play an important role in the developing TME^[75–77]^. A hallmark of senescent cells is the secretion of a wide variety of growth factors, pro-inflammatory cytokines, chemokines, and proteinases, a characteristic termed the senescence-associated secretory phenotype (SASP) [Figure 1]^[78,79]^. Under normal conditions, the SASP-factors act in an autocrine manner to maintain the senescence program and recruit immune cells into the local environment^[80–83]^. However, paracrine signaling by SASP components can also influence the behavior of adjacent cells, engaging signaling programs that contribute to tumor progression and therapy failure^[64,84–89]^. A collection of recent studies has demonstrated the ability of senescent cells and SASP components in the TME to drive cellular E-M plasticity and the expansion of a CSC-like cell population^[90,91]^. In fact, the SASP program can promote stemness within both senescent cells and neighboring cells, both *in vitro* and *in vivo*, through secretion of potent inflammatory cytokines associated with disease recurrence, and overall poor prognosis^[92,93]^. More specifically, less aggressive luminal MCF-7 cells were treated with conditioned medium harvested from senescent populations experiencing SASP. Exposure to conditioned media led to a more CD24LO/CD44HI invasive/stem like population similar to already aggressive MDA-MB-231 cells, which was dependent upon IL6 and IL8, two well defined SASP-factors, secretion^[94]^. Furthermore, sustained hyper-activation of signal transducer and activator of transcription 3 (STAT3) by SASP components plays a critical role in induction of an invasive and stem-like program^[95]^. Taken together, the presence of malignant, pre-malignant, and senescent epithelial cells creates a diverse TME suitable to drive E-M/CSC plasticity within the tumor and contribute to metastatic, therapy-resistant, and tumor-initiating phenotypes. Below we will discuss how E-M/CSC plasticity contributes to these deadly phenotypes responsible for patient mortality.

## AN IDENTITY CRISIS: MESENCHYMAL *VS*. EPITHELIAL

Each step along the metastatic cascade presents a new environmental context and challenge that a potentially metastatic cell must adapt to in order to thrive. This adaptation involves changes in a cell’s state. Cellular plasticity is defined as the ability of a cell to acquire new biological properties due to intrinsic and extrinsic cues. It is important to recognize that plasticity is most often a highly dynamic and reversible process that can be used to describe multiple cellular changes (i.e., differentiation, metabolism, response to immune cells, motility, and cell fate). Throughout this review, we will refer to cellular plasticity as the ability for cells to undergo E-M/CSC plasticity, that is, cells shifting between epithelial/non-CSC and mesenchymal/CSC states. E-M/CSC plasticity is important in imparting invasive and motile phenotypes as well as cells acquiring tumor-initiating potential and reduced sensitivities to therapy.

The switch from epithelial to mesenchymal state facilitates dissolution of cell-cell junctions (due to the repression of cell adhesion proteins E-Cadherin, EPCAM, and CD24) and increased ability to remodel the surrounding ECM [due to matrix-metalloprotease proteins (MMP), adamalysins (ADAMs), and differential integrin expression]^[3,96–101]^. The corresponding cytoskeletal and ECM remodeling imparts a migratory and invasive phenotype important for metastasis. Lineage tracing experiments confirm that epithelial-to-mesenchymal transition (EMT) occurs *in vivo*, and that it precedes metastasis in murine BC models^[102–104]^. Through intravital imaging, Beerling and colleagues showed that mesenchymal tumor cells have a unique and specific migratory behavior that results in greater circulating tumor cells (CTC), increased tumor cells within the lungs, and metastasis; in contrast, the more abundant epithelial cell population remained non-motile and less metastatic^[104]^. It should be noted that others, Zheng *et al*.^[105]^ and Fischer *et al*.^[106]^ have employed *in vivo* lineage tracing models and reported that EMT is not required for metastasis. As Beerling and colleagues discuss, many of these reports rely on fixed gene manipulation (for example, gene silencing or protein overexpression) to experimentally test an EMT-underlies-metastasis hypothesis. It is possible that such artificial manipulation is not able to recapitulate physiologic events and, in this way, contributes to discrepancies in findings. Other small, but crucial, details could play a further role in some discrepancies: (1) EMT may be indispensable to metastasis for select cancer subtypes, but dispensable for others; (2) reliance on activation of a single gene reporter (e.g., Fsp1) to capture and “tag” an EMT event restricts the sensitivity of the model system; (3) criteria for how the EMT program is identified, such as the panoply of specific “epithelial” or “mesenchymal” proteins that are induced or suppressed, may also lead to false-negatives if these identifying protein sets are incongruent across cancers and cancer subtypes. Regarding the latter point, Zheng *et al*.^[105]^ and Fischer *et al*.^[106]^ reported on Vimentin and E-cadherin status, but each represents just one exemplar for mesenchymal or epithelial cell state and ultimately, these may not be the most relevant. Finally, evidence that metastases occur in the absence of EMT does not preclude a potential for EMT to enhance cancer cell metastasis.

The gain of migratory and invasive properties which accompany E-M plasticity occurs concomitantly with changes in global signaling programs and gene expression. Several key transcription factors (TF) have been identified as master regulators of EMT: SNAI1 (Snail), SNAI2 (Slug), ZEB1, ZEB2, TWIST1, and TWIST2^[107–112]^. These TF are typically kept silenced in adult cells when plasticity is unnecessary but become aberrantly activated by TME factors to induce EMT^[113,114]^. EMT-TF expression strongly correlates to regions of the tumor with mesenchymal marker positivity, notably, the invasive front of the tumor where mesenchymal cells act as ‘trailblazers’ that initiate and guide local metastasis^[108,115–118]^.

Tissue-invasive cells that encounter vessels may intravasate into vascular or lymph networks and be disseminated throughout the body as CTC. CTC are not only present in measurable quantities in patients with BC, but their abundance is predictive of overall survival and directly correlates with the likelihood of relapse following treatment^[119–123]^. Moreover, CTC display a wide range of markers associated with cells that have or are undergoing E-M/CSC reprogramming. CTC often exhibit a decrease in epithelial markers CD24, E-Cadherin (CDH1), EPCAM and an increase in well-known mesenchymal and CSC markers (ZEB1, Snail, CD44, Vimentin)^[116,124–127]^. Critically, CTC are capable of tumor initiation at a secondary site, and more importantly demonstrate remarkable plasticity by engaging specific molecular programs that dictate the organ-specificity of metastases^[128–133]^. Overall, mesenchymal cells are simply more well-suited to the task of escaping the primary site and reaching distant organs.

However, escaping the primary site and circulating throughout the lymph or cardiovascular system is not sufficient for metastases to develop. Once a mesenchymal cell has reached a secondary organ, it must embed itself in the new tissue and flourish in order to establish a metastatic outgrowth; not all cells have this capability^[3,134]^. CSC have been deemed the “roots” of primary and secondary site outgrowth due to their tumor-initiating capacity and their ability to differentiate into multiple lineages, recreating the heterogeneity seen in the primary site^[135–137]^. Since Al-Hajj *et al*.^[138]^’s initial isolation of CSC from BC models, marked by CD44HI/CD24LO cell surface expression profile, the field has made significant insight into the CSC paradigm^[138]^. Shortly after, Ginestier *et al*.^[139]^ demonstrated an increase in aldehyde dehydrogenase 1 (ALDH1) activity strongly correlated with both normal and malignant stem/progenitor cells within BC. CSC have been isolated from nearly every human cancer through identification of surface marker expression of nearly 40 different markers which vary from cancer to cancer (CD133, CD44, CXCR4, CD90, *etc*.)^[140]^. Further complicating matters, our understanding of the dynamics of breast cancer stem cell (BCSC) populations is poorly understood. For instance, Liu *et al*.^[141]^ identified ALDH1 activity to inversely correlate with CD44 expression in BC models, suggesting a complex make up of phenotypes contributing to tumor initiation, which further work is required to better understand. Regardless of differences in marker expression, isolated CSC populations all exhibit similar characteristics in terms of their ability to initiate tumor formation in limiting dilution, produce multiple cell lineages, maintain tumor-initiating potential through periods of metastatic latency, and survive cytotoxic therapies due to a wide range of resistance mechanisms^[142,143]^. Seminal papers from the Weinberg and Puisieux groups provided the first evidence of a link between shifting from epithelial to mesenchymal identity and the acquisition of CSC properties in BC^[144,145]^. Prior to this, the existence of CSC and the occurrence of EMT in malignant neoplasms had each been garnering significant attention, but an association between the two phenotypes had yet to be demonstrated. Specifically, induction of EMT led to a shift in surface marker profiles from CD24HI/CD44LO to CD24LO/CD44HI, resulting in (1) the ability to generate tumorspheres and tumors (self-renewal potential); and (2) the ability to give rise to differentiated daughter cells^[144–148]^.

**Figure 2.**
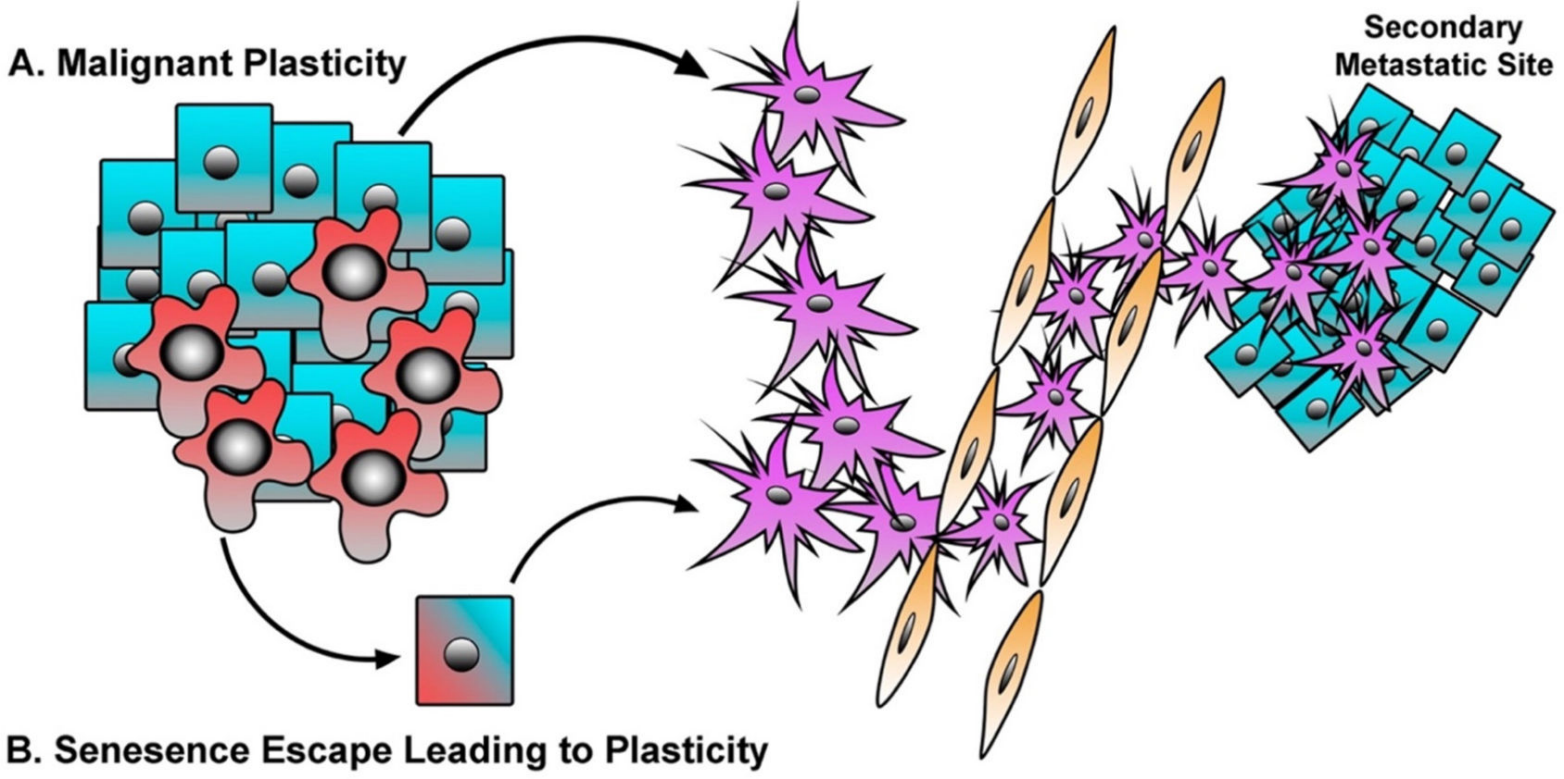
Malignant and Senescent Epithelial Cells Contribute to Metastasis. A: malignant cells respond to TME cytokines or intrinsic genetic alterations in order to drive E-M/CSC plasticity, resulting in greater metastasis, enhanced disease recurrence, and therapeutic resistance; B: pre-malignant cells undergo senescence in response to aberrant oncogene or cytokine signaling. However, in some instances a small population of senescent cells may undergo E-M/CSC plasticity, and thus escape the tumor suppressive barrier of senescence and further contribute to the metastatic phenotype

One implication of this finding is that breast CSC may not solely arise through malignant transformation of a pre-existing ‘normal’ mammary stem cell. More recent work, including ours, has described the ability for transformed epithelial/non-CSC populations to acquire mesenchymal/CSC properties in response to autocrine or paracrine signaling initiated from secreted factors within the TME. Importantly, the ability to fluidly move between epithelial/non-CSC and mesenchymal/CSC states has been shown to facilitate metastatic outgrowth [Figure 2A], as cells are believed to reactivate a proliferative program and reacquire epithelial cell phenotypes at the site of metastasis^[12–14,137,141,149–163]^.

## THE QUIET NEIGHBOR: PRE-MALIGNANT CELLS AND PLASTICITY

E-M/CSC plasticity is a major contributor to tumor heterogeneity, progression towards metastasis, and therapy failure. Yet, cellular plasticity also plays a crucial role in normal physiology, such as embryonic development, wound healing, and tissue remodeling^[164,165]^. For instance, differentiated epithelial cells can dedifferentiate or trans-differentiate, wherein a differentiated cell may change state into an alternate lineage. These forms of plasticity are seen routinely in skin cells, hepatocytes, colon epithelium, pancreatic acinar cells, and others as they undergo a phenotypic transition in order to repair and sustain the homeostatic nature of the tissue^[166–170]^. In fact, cellular plasticity of mammary epithelial cells has been suggested to be a driver of the heterogeneity seen in BC^[171–173]^.

Similarly, normal mammary gland organogenesis and homeostasis requires the presence of mammary stem cells (MaSCs) in the mammary gland epithelium that differentiate into both progenitor cells and differentiated cells^[174]^. Remarkably, many of the markers used to distinguish normal MaSCs overlap with those used to identify breast CSC including mRNA expression profiles^[174]^. Interestingly E-M plasticity is also essential for normal mammary gland development and maintenance. For example, E-M plasticity is crucial for MaSC migration as well as the expansion and invasion of terminal end buds (TEB) during mammary gland development^[174]^. Much like the mechanisms that guide stemness in MaSC, the upregulation of multiple EMT-TFs, such as Snail and Twist, was uncovered in the microenvironment of the TEB by genomic transcriptional analysis^[175]^. E-M plasticity in non-MaSC cells that comprise the normal mammary gland guides cell polarity and the organization of mammary cells into their well-defined glandular structures^[176]^. Given its importance in tissue remodeling and homeostasis, the normal processes governing cellular plasticity are strictly regulated^[177–179]^.

As discussed above, senescence serves as an important tumor suppressive barrier to prevent transformation and the outgrowth of a dysregulated and uncontrolled population. Interestingly, the induction of senescence in normal cells often results in the simultaneous emergence of mesenchymal/stem-cell markers in conjunction with senescence markers, and loss of proliferative capacity. For example, senescence leads to the induction of CD44 expression, a cell surface marker regularly used to distinguish breast CSC from non-CSC, and often expressed as malignant cells acquire MES/CSC properties and undergo plasticity^[138,180–183]^. As small subsets of senescent cells dismantle the senescence program, the emergent population of proliferating cells can harbor a mesenchymal/stem-cell phenotype that can persist throughout the remainder of the transformation process, ultimately yielding a more invasive and aggressive cancer cell^[184–187]^. Li *et al*.^[15]^ demonstrated that many of the transcriptional changes observed in BC are also initiated in normal HMEC as they escape senescence. In their study, HMEC were shown to have a “pre-transformation” transcriptome and exhibited a partial EMT following their senescence escape^[15]^. Similarly, normal HMEC that have spontaneously escaped replicative senescence exhibit a greater mesenchymal and CD24LO/CD44HI CSC-phenotype^[16,188]^. Further studies have suggested cell cycle regulator Cyclin A1 and tumor suppressors p53 and p16 can act as “gatekeepers” to maintain cells in an epithelial state. Dysregulation of these proteins in epithelial cells can result in the initiation of the mesenchymal/stem-cell program, which interestingly corresponds with the escape from senescence^[189,190]^. Moreover, induction of EMT (via Snail, Twist, and ZEB1 expression) prior to a cell’s engagement of oncogene-induced senescence, prevents senescence altogether and results in the induction of a CSC program and tumor initiation^[191–196]^.

In addition to pre-malignant cells, cytotoxic therapies can drive cancer cells into a therapy-induced senescence (TIS). Again, cells that escape TIS have acquired a senescence-associated stemness^[197–199]^. Milanovic *et al*.^[200]^ have shown that cells undergoing TIS express a variety of stem-cell associated markers, and that TIS in B-cell lymphomas exhibit a gene signature which mirrors that of adult tissue stem cells, conferring a highly aggressive phenotype responsible for tumor relapse. The emergence of a stem-like population following the engagement of senescence may be an inherent phenomenon that also occurs during other stress-induced senescence responses (i.e., oncogenes, replication stress, γ-irradiation) among different cell types^[200–202]^. Altogether, these findings suggest that, the mesenchymal/stem-cell program engaged during senescence may have significant negative consequences if those cells can overcome the signals maintaining senescence, resulting in cells with greater ability metastasize and survive therapy [Figure 2B]. Thus, we suggest that therapies that target senescent cells would limit the reservoir of aggressive cells harboring a senescence-associated stemness responsible for therapy failure and relapse. In the following section we will discuss the potential of targeting E-M/CSC plasticity within malignant, pre-malignant, and senescent populations.

## HALTING METASTASIS: TARGETING PRE- AND POST-MALIGNANT CELL PLASTICITY

Liquid biopsies from patients with early-stage BC receiving neoadjuvant therapy can be used to identify subjects at high risk of recurrence by quantifying the number of CTC. Furthermore, expression of mesenchymal markers in the CTC correlates with poor prognosis and response to therapy^[138,203–206]^. With the advent of single cell analysis techniques, our understanding of the evolution and diversity of tumor cells that are responsible for invasion, metastasis, and therapy failure is expanding. For example, single-cell qPCR has identified mesenchymal/CSC gene expression patterns in early-stage breast cancer micro-metastases^[137]^. In contrast, later-stage metastases (from the same PDX tissue) are more heterogeneous, more proliferative, express differentiation markers, and display greater similarity to the primary tumors. The findings are consistent with the idea that mesenchymal/CSC initiate metastatic outgrowth at a secondary site, followed later by increased proliferation and differentiation. More recent single cell RNA-sequencing (scRNA-seq) studies have confirmed that EMT in primary tumors proceeds through distinct, hybrid states, ranging from completely epithelial to completely mesenchymal^[207]^. These E-M hybrids, which harbor the greatest level of plasticity, are more efficient at intravasating, extravasating to the lungs, and forming metastases^[208]^. Underlying this biology, E-M hybrids have distinct chromatin landscapes and transcriptional profiles. *In situ* analysis identified increasing vascularization and immune cell infiltration (particularly macrophages) nearest the E-M hybrids and fully mesenchymal cells^[208]^. A separate scRNA-seq study determined that, in response to chemotherapy, emerging chemo-resistant cells undergo transcriptional changes consistent with EMT. In most patients, this chemo-resistant transcriptional program was not evident before treatment but acquired via transcriptional reprogramming following treatment^[209]^. These studies and others make a strong case that epithelial tumor cells can be induced into a drug-tolerant, E-M hybrid cell state by chemotherapy^[141,209–214]^.

Identifying and targeting the pathways responsible for this chemo-resistant reprogramming would help improve the efficacy of chemotherapy. In a recent example, SRC kinase inhibition prevented the *de novo* generation of chemo-resistant cells^[209]^. Importantly, this chemo-sensitization was temporally dependent, and only effective if SRC inhibition occurred after chemotherapy, when the signaling responsible for generating the chemo-resistance phenotype had become activated. More recently, Cazet *et al*.^[215]^ identified crosstalk between TNBC models and cancer-associated fibroblasts (CAF), which promoted stemness and drug resistance in the cancer cells via paracrine Hedgehog (Hh) signaling. A key developmental pathway, Hh signaling requires receptor mediated binding of Hh to Patched (PTCH), resulting in Smoothened (SMO)-mediated nuclear translocation of Gli1. Gli1 then acts as a transcription factor, mediating Hh pathway transcriptional changes^[216]^. Hh signaling is often reactivated in a subset of BC, and specifically in the context of TNBC, a paracrine manner^[217]^. After targeting paracrine Hh signaling *in vivo*, via two clinically available smoothened inhibitors (SMOi) Vismodegib and Sonidegib, Cazet *et al*.^[215]^ observed a suppression of cancer cell plasticity and increased sensitivity to docetaxel. Most importantly, in a phase I clinical trial, 3 of 12 patients with metastatic TNBC observed clinical benefit from combinatorial therapy of SMOi and docetaxel (one with complete response), similar to treatment paradigms we suggest above. SMOi have been beneficial for basal cell carcinomas and medulloblastomas where tumors rely on cell-autonomous hedgehog signaling, however the work here suggests the ability to target the TME in order to dampen cancer cell plasticity, and achieve a greater therapeutic response^[216,218,219]^. Below we will address additional potential therapeutic avenues one may use to dismantle E-M/CSC plasticity in order to prevent metastatic dissemination, secondary site outgrowth, or re-sensitize cancer cells to standard of care therapies.

## TARGETING MALIGNANT POPULATIONS

### STAT3

STAT3 is persistently activated in cancer cells, as it is a downstream effector of several receptor tyrosine kinases (RTKs) commonly activated by growth factors and cytokines^[220–222]^. We, as well as others have demonstrated that, persistent STAT3 activation in cancer cells induces mesenchymal and CSC properties, inhibits apoptosis, and maintains a more un-differentiated phenotype^[12,14]^. Therefore, STAT3 is a promising therapeutic target. A number of small-molecule inhibitors of STAT3 (KI16; BP-5–087; WP1066) are currently in development and combination therapies, with BCR-ABL1 or BRAF inhibitors, have shown positive results in the treatment of several cancer types^[223–225]^. In the context of TNBC, a recent phase Ib/II study combining the cancer stemness inhibitor Napubacasin (BB608), which prevents STAT3 activation, with weekly administrations of paclitaxel showed improvement in metastatic patients whose cancer had progressed while on a taxane-based regimen (NCT01325441).

### PI3K, Akt and mTOR

The PI3K/Akt/mTOR pathway is involved in several cell processes, including proliferation, metabolism and motility, therefore it is not surprising that its dysregulation corresponds to uncontrolled proliferation and propagation in a wide spectrum of cancers. The role of the PI3K/Akt/mTOR pathway in maintaining cell plasticity in cancer has been documented in several publications^[226–228]^. In the context of breast cancer, *PIK3CA* mutations have been observed in each of the different subtypes, but mostly in hormone receptor-positive tumors where it’s associated with disease progression and resistance to endocrine therapy. Each *PIK3CA* mutation results in an abnormal activation of the alpha subunit of PI3K, that with the beta subunit is the most common in breast tissue^[229]^. *PIK3CA* mutations appear to hold prognostic and predictive value in hormone receptor-positive, HER2-negative advanced or metastatic breast cancer. Several studies show how targeting tumors carrying a *PIK3CA* mutation with *PIK3CA* inhibitors increased the PFS of patients^[230]^. In January 2019, results from the phase III SANDPIPER clinical trial (NCT02340221) were posted. This international, multicenter, randomized, double-blinded, placebo-controlled study was designed to test the efficacy of a combo of the *PIK3CA* SMI taselisib and the synthetic estrogen receptor antagonist fulvestran versus placebo and fulvestran in the treatment of ER-positive, HER-2-negative locally advanced or metastatic breast cancer harboring a *PIK3CA* mutation in patients with disease recurrence after or during treatment with aromatase inhibitor (AI). SANDIPIPER is the first placebo-controlled trial testing the efficacy of a mutant-specific PI3K inhibitor^[231]^. Taselisib is specifically directed against the alpha isoform of *PIK3CA*, however it can inhibit the gamma and delta isoforms as well, thus causing an increase in toxicity mostly involving the gastro-intestinal tract. Another phase III clinical trial, BELLE-2 (NCT01610284) is analyzing the effects of the pan-PI3K inhibitor buparlisib in combination with fulvestran compared to fulvestran and placebo combo. Unfortunately, also buparlisib showed important side effects, particularly hyperglycemia^[229]^. Several other PI3K inhibitors are currently under investigation in clinical trials, including apelisib (NCT02437318 - SOLAR-1), which showed encouraging clinical benefits in the majority of patients enrolled^[232]^. In the future, research efforts should be more focused at inhibiting exclusively the alpha subunit of *PIK3CA*, thus reducing the risk of toxicity and side effects. Interestingly, GDC-0077 from Genentech appears to be extremely more specific towards the alpha subunit of PIK3A over other subunits, thus representing a potentially less toxic alternative to other inhibitors and is currently investigated in a phase I clinical trial alone or in combination with other agents, such as palbociclib, letrozole and fulvestran (NCT03006172).

While the role of mTOR signaling in promoting a CSC phenotype is still controversial, its activation in BC appears to be essential for colony formation *in vitro* and tumorigenicity^[233]^. Furthermore, mTOR signaling increases aldehyde dehydrogenase 1 (ALDH1) activity^[234,235]^. IGF-1R activation signaling through PI3K/Akt/mTOR represents a promising target in BC as an abnormal activation of PI3K can also lead to an increased activation of STAT3 through enhanced expression of the chemokine (C-X-C motif) receptor 4 (CXCR4)^[236,237]^. IGF-1R is activated in the 50% of breast cancer patients. Several phase III clinical trials targeting IGF-1R with small molecule inhibitors have so far failed due to side effects such as hyperglycemia and metabolic syndrome caused by the homology of IGF-1R and insulin receptor (IR) and IGF elevation in response to disruption of glucose homeostasis. On the other hand, monoclonal antibodies specifically targeting IGF-1R or its ligand have shown a higher specificity by sparing the insulin metabolism from any inhibitory effect in preclinical models^[238]^. Early phase trials have been setup to determine the efficacy of an antibody-based inhibition of IGF-1R signaling by targeting either the receptor or the ligand in combination with aromatase inhibitors or mTOR inhibitors but with small success^[239]^. A more complex therapeutic protocol combining inhibitors of PI3K or other downstream effectors of IGF-1R would probably be more beneficial.

Several drugs targeting the PI3K/mTOR pathway have shown a selective effect on CSCs, inhibiting their growth and sensitizing them to traditional chemotherapy. Since 2007, when the Food and Drug Administration (FDA) approved the mTOR inhibitor temsirolimus for the treatment of advanced renal carcinoma, new generations of compounds have been developed. Everolimus, is a dual PI3K/mTOR inhibitor, which blocks all PI3K class I isoforms as well as mTORC1 and 2, thus preventing the development of CSCs when combined with letrozole^[240,241]^. Interestingly, mTOR is also inhibited by metformin (1,1-dimethylbiguanide hydrochloride), usually prescribed for the treatment of type 2 diabetes. Metformin preferentially kills CSCs over non-CSCs and reduces tumorsphere formation and CSC marker expression (CD133, CD44 and ALDH1)^[242]^. Similar effects have been achieved with the antibiotic salinomycin, which selectively kills breast CSC^[243]^. The plant-derived chemotherapeutic molecule rottlerin induces apoptosis in breast CSC by inhibiting PI3K/Akt/mTOR pathway^[244]^. More recently, the PI3K/mTOR dual inhibitor VS5584 has shown promising results by delaying tumor recurrence through selective killing of CSC after chemotherapy^[245]^.

### Notch

Notch signaling is known to be involved in different cellular processes, including differentiation and proliferation. Perturbation in these processes can be caused by mutations in Notch or one of its effectors. Mutations of the Notch pathway are the hallmark of many subtypes of cancer, including BC, where cells overexpressing Notch have increased CSC markers (SOX2 and OCT4) and phenotypes (tumorsphere formation)^[246–248]^. Moreover, Notch promotes EMT and metastasis in TNBC cells and Notch inhibition can prevent EMT both *in vitro* and *in vivo*^[249]^. For example, 3,6-dihydroxiflavone (3,6-DHF) causes a significant reduction of CSCs *in vitro* and blocks lung metastasis by specifically down regulating Notch, Hes-1 and c-Myc^[250]^. 3,6 DHF shows potent chemo-preventive properties against breast carcinogenesis both *in vitro* and *in vivo*, although a mechanism has not been identified yet, besides an epigenetic increase in the synthesis of miR-34a, a potent down-regulator of Notch and thus of EMT in breast cancer^[251]^. Furthermore, 3,6-DHF down-regulates the expression of Notch’s target genes *Hes1*, c-*Myc* and the EMT mediators SNAIL, Twist and Slug by compromising the formation of the transcriptional complex NICD-CSL-MAML^[252,253]^. More recently, it has been shown that Notch3 inhibition by siRNA silencing increases TNBC sensitivity to gefitinib in EGFR-Tyrosine kinase inhibitors (TKI)-resistant cells by blocking the nuclear translocation of activated EGFR^[254]^.

### Wnt and β-catenin

The Wnt pathway is involved in the maintenance of breast CSCs by promoting self-renewal and plasticity through PAF (proliferating cell nuclear antigen-associated factor)^[255]^. The Wnt ligand, Frizzled, is upregulated in high-grade tumors, including more aggressive forms of BC, and can cause EMT and metastasis through non-canonical STAT3 activation^[256]^. Inhibitors targeting the Wnt pathway have been developed and are showing promise in BC models. In particular, the Wnt/beta-catenin inhibitor CWP232228, which blocks beta-catenin binding to T-cell factor (TCF) in the nucleus, prevents the proliferation of breast CSC, selectively depleting CD133-positive and ALDH1-high cells both *in vitro* and *in vivo*^[257,258]^.

## TARGETING SENESCENT POPULATIONS

As described in the previous section, cells escaping senescence exhibit increased invasive and tumor-initiating properties. On top of that, senescent cells secrete a variety of cytokines and growth factors as part of the SASP. The production of these factors into the TME has been shown to drive E-M/CSC plasticity in neighboring cells, as well as the senescent cells themselves, expanding the population that can facilitate metastasis and drug resistance. Moreover, the chronic presence of senescent cells can impair local tissue function, create a highly inflammatory environment, and in some instances exacerbate the side effects of chemotherapies^[259,260]^. Considering conventional treatments such as chemo- and radio-therapy often induce senescence in both cancer and normal cells, it seems pertinent to target senescent cells and clear them from the local tissue. The concept for targeting senescent cells was brought to light by Lee *et al*.^[261]^ by exploiting the high lysosomal β-galactosidase activity in senescent cells, cytotoxic drugs encapsulated in galacto-oligosaccharides particles (galNP beads) can target chemotherapy-induced senescent cells in mice^[262]^. Preclinical results showed a significant regression of tumor xenografts after treatment with galNP beads loaded with doxorubicin in combination with palbociclib^[263]^. Moreover, senescent-cell accumulation in mice can be reduced by treatment with potential senolytic agent, Navitoclax (ABT-263), a small molecule inhibitor of the anti-apoptotic proteins BCL-2 and BCLxL^[264,265]^. However, targeting senescent cells is a relatively new concept, and further insights into the signaling mechanisms which senescent cells rely on is needed.

## CONCLUSION

To date our ability to target BC metastases have been largely unsuccessful. With patient survival falling to 22% for those that reach distant and wide-spread disease, the ability to target cells at various stages of the metastatic cascade is greatly needed. Here, we have focused on an epithelial cancer cell’s ability to out-maneuver cytotoxic agents by changing cell state; E-M/CSC plasticity. This induced reprogramming often reduces sensitivity to therapy by a number of mechanisms, creating an immense problem with effectively removing the disease. We propose to remove the molecular “escape hatch” which provides cells that have undergone E-M/CSC reprogramming a sustained advantage in survival and resurgence. An approach that combines readily available small molecule inhibitors of plasticity-inducing pathways in conjunction with commonly used front-line therapies should increase therapeutic sensitivities. Often, pathway-selective small molecule inhibitors that make it to the clinic are used with the objective of inducing growth inhibition or cell death. However, many of these inhibitors may present a wide range of side effects by acting on nontumor cells or show little efficacy as a single agent^[266–268]^. Instead, a low dosage may prevent toxicity or off-target effects while reducing a tumor cells ability to undergo reprograming to a more MES/CSC-like state. In doing so, these combination therapies may overcome the MES/CSC-like programs which promote therapy failure and metastatic disease progression, ultimately rendering populations sensitive to currently used chemotherapies and increasing overall patient survival.

Cell plasticity is garnering significant interest in the field of cancer biology, as we attempt to better understand the metastatic process, and those drivers behind it. However, we do not yet fully understand what allows an epithelial cell to undergo MES/CSC reprograming or what pathways maintain this newly attained phenotype. While most of the work understanding plasticity has been done on malignant populations, recent studies have hinted at the ability of pre-malignant populations to undergo MES/stem-cell reprograming, albeit often halted by intact tumor suppressive mechanisms, leading to senescence. Here, we discussed the ability of pre-malignant epithelial cells to undergo a MES/stem-cell reprogramming that is comparable to that observed with malignant cells. However, much remains to be discovered about the importance of these pre-malignant populations. We still do not yet know which cells within a pre-malignant population have escaped the senescence “barrier”, which in turn generate more aggressive sub-populations capable of driving metastasis. Identifying, or molecularly defining, which cells possess the capability to escape senescence may allow us to predict the aggressiveness of a patient’s metastatic burden and open potential avenues for targeting cells with the potential to escape senescence. Ultimately, targeting the signaling responsible for plasticity, both in pre-malignant and fully transformed cells, will enhance the efficacy of chemotherapies and suppress a key driving force responsible for patient death.
